# Cervical gunshot injury with esophageal perforation and bullet migration to the stomach: a rare case report

**DOI:** 10.1097/RC9.0000000000000563

**Published:** 2026-06-03

**Authors:** Reza Hosseinpour, Sepehr Moradi, Taha Negahdari

**Affiliations:** a Department of Surgery, Breast Surgery Division, Yasuj University of Medical Sciences, Shahid Jalil Hospital, Yasuj, Iran; b Department of General Surgery, Student Research Committee, Shiraz University of Medical Sciences, Shiraz, Fars, Iran; c Department of Medical Education (Medical Internship), Yasuj University of Medical Sciences, Yasuj, Iran

**Keywords:** bullet migration, case report, esophageal perforation, penetrating neck injury, trauma surgery

## Abstract

**Background:**

Penetrating neck injuries (PNIs) are uncommon but are associated with considerable morbidity and mortality, with reported rates approaching 10% due to the concentration of vital structures in the cervical region. Esophageal injuries – often initially missed – are a major contributor to delayed mortality. Prompt assessment of the airway, hemodynamic stability, and associated injuries is essential. Here, we report a rare case of a previously healthy patient who sustained a PNI with a subsequent delayed presentation of esophageal perforation.

**Case presentation:**

A 17-year-old male presented 3 hours after sustaining a Zone II gunshot wound to the right side of the neck. He was hemodynamically stable, and initial imaging showed no abnormalities. Twelve hours later, the development of fever prompted further evaluation with abdominal computed tomography (CT), which revealed the projectile in the stomach, suggesting migration through the esophagus.

**Discussion:**

Even hemodynamically stable patients with PNIs may develop delayed complications, including esophageal perforation and bullet migration, which can be missed during the initial assessment. Recognition of evolving symptoms, early repeat imaging, and timely surgical intervention are essential in achieving favorable outcomes.

**Conclusion:**

PNIs require vigilant monitoring and a structured, guideline-based diagnostic approach to avoid missed aerodigestive injuries. To our knowledge, this is the first reported case of a cervical gunshot projectile migrating into the stomach through the esophagus.

## Introduction

Penetrating neck injury (PNI) is defined as trauma in which a projectile or sharp object breaches the platysma muscle, extending from the skull base superiorly to the sternum inferiorly^[^1^]^. Although PNIs account for a relatively small proportion of traumatic injuries, they are associated with significant morbidity and mortality, with reported death rates of approximately 10%^[^1^]^. This heightened risk is due to the dense concentration of vital structures in the neck, including major vascular, airway, digestive, and neurologic systems. Among these, esophageal injuries are particularly important, as they represent a major cause of delayed mortality and frequently remain undetected during the initial evaluation^[^2^]^.

Most esophageal injuries result from penetrating trauma, with gunshot wounds responsible for 70–80% and stab wounds accounting for an additional 15–20% of cases. Blunt mechanisms are rare, comprising fewer than 1% of all esophageal injuries^[^3^]^.

HIGHLIGHTS

- This case report presents the first documented instance of a cervical gunshot projectile migrating through the esophagus into the stomach.
- Despite hemodynamic stability and unremarkable initial imaging, the patient developed delayed esophageal perforation, underscoring the diagnostic challenges in penetrating neck injuries.
- The case demonstrates the importance of repeated imaging and close clinical monitoring when initial studies fail to identify aerodigestive injuries.
- Successful surgical repair using a sternocleidomastoid muscle flap resulted in full recovery, highlighting an effective operative strategy for cervical esophageal perforation.
- This report emphasizes adherence to structured trauma protocols to prevent missed or delayed diagnoses in penetrating neck trauma.

Effective management of PNI requires rapid intervention, thorough airway and circulatory assessment, and evaluation for skeletal and neurologic involvement^[^2^]^. In the present case, despite a clear projectile entry wound in the right lateral neck (Fig. 1), the patient exhibited no early signs of esophageal or airway involvement, including no dysphagia, odynophagia, hematemesis, hemoptysis, or subcutaneous emphysema, during the initial clinical evaluation. The wound was limited to a single, non-actively bleeding entry site measuring approximately two fingertip widths. According to the patient, the injury resulted from a close-range civilian altercation involving a handgun, although the bullet caliber was unknown.

**Figure 1.**
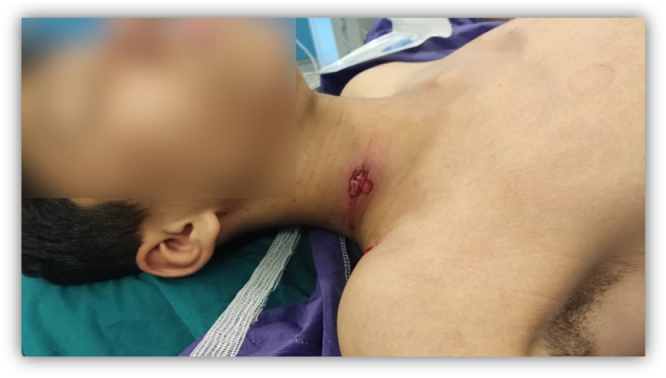
Projectile entry site.

Although cervical penetrating gunshot wounds with esophageal perforation have been reported, the migration of the projectile into the stomach appears to be exceedingly rare. We present this case to emphasize the possibility of delayed aerodigestive injury and to highlight important diagnostic considerations in neck trauma. This article is structured in accordance with the 2025 SCARE guideline^[^4^]^.

## Case presentation

A 17-year-old male presented to the emergency department approximately 3 hours after sustaining a gunshot wound to the right lateral neck (Zone II) during a street altercation (Fig. 1). On arrival, he was alert, communicative, and hemodynamically stable. His initial vital signs included a blood pressure of 120/80 mmHg, a heart rate of 75 beats per minute, a respiratory rate of 20 breaths per minute, a temperature of 37°C, and a Glasgow Coma Scale (GCS) score of 15. A complete ABCDE assessment was performed in accordance with Advanced Trauma Life Support (ATLS) principles.

Physical examination revealed a single, well-defined projectile entry wound on the right side of the neck, measuring approximately two fingertip widths. No external active bleeding was present, and only mild localized tenderness was noted. There was no evidence of neurologic deficit, hoarseness, stridor, subcutaneous emphysema, hematemesis, hemoptysis, dysphagia, or odynophagia. No respiratory or digestive compromise was detected.

Initial laboratory investigations – including complete blood count and serum biochemistry – were unremarkable. The patient reported sustaining the injury at close range with a civilian handgun during an altercation, although the bullet caliber was unknown.

Initial imaging was performed in sequence and consisted of lateral and anteroposterior (AP) neck radiographs, followed by head and neck computed tomography angiography (CTA), and subsequently a barium swallow study (Fig. 2). All studies demonstrated normal findings, with no vascular injury, no aerodigestive tract disruption, and no evidence of contrast leak on the swallow examination. Despite these evaluations, the bullet remained undetected. The patient stayed clinically stable and continued to tolerate oral intake throughout observation.

**Figure 2.**
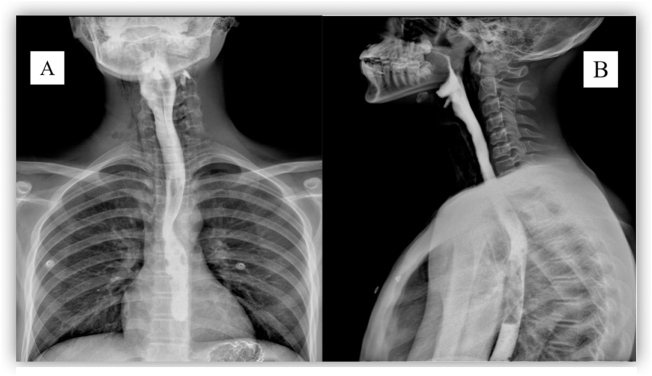
Barium swallow. (A) showing AP view and (B) showing lateral view.

Approximately 12 hours after admission, he developed an isolated fever without additional symptoms. Given the new-onset fever and the unexpected identification of a high-density projectile within the stomach on abdominal CT, delayed esophageal perforation with bullet migration was suspected, leading to the decision for surgical exploration. The abdominal CT demonstrated a round metallic foreign body within the stomach, consistent with migration through the esophagus (Fig. 3).

**Figure 3.**
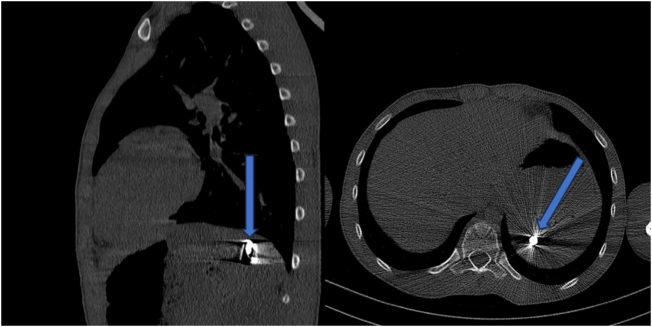
High density foreign body consistent with bullet migration through the esophagus to the stomach. Sagittal view and axial view. Two arrows indicating the position of the bullet in the CT scan imaging of the stomach.

The patient underwent surgical exploration via a left cervical incision along the sternocleidomastoid (SCM) muscle. The bullet tract was identified, revealing a 10 × 10 mm perforation of the cervical esophagus. The defect was repaired and reinforced with an SCM muscle flap to ensure adequate vascular coverage. A Penrose drain was placed for postoperative drainage.

Postoperatively, the patient received intravenous vancomycin and meropenem, along with intramuscular pethidine for analgesia. His postoperative course was uneventful. During the postoperative observation period, the bullet was neither retrieved surgically nor removed endoscopically. Follow-up abdominal radiography and CT performed several days later showed no remaining foreign body within the gastrointestinal tract. These findings suggested that the bullet had passed through the gastrointestinal system and was expelled spontaneously in the stool. The patient was discharged in good condition after 7 days of inpatient care.

## Discussion

We report the case of a 17-year-old male who sustained a penetrating cervical gunshot wound that resulted in esophageal perforation and subsequent migration of the projectile into the stomach – an exceedingly rare trajectory. While cervical esophageal injuries from gunshot trauma have been reported, the passage of a bullet from the neck into the stomach through the esophagus appears to be very uncommon in the literature^[^5–7^]^.

Early imaging may miss small or evolving cervical esophageal perforations. Contrast esophagography can have false-negative rates of up to 10–40% in small or contained injuries^[^8^]^, and fluoroscopic swallow studies may fail to detect subtle leaks^[^9^]^. CTA is also limited in identifying early mucosal defects^[^10^]^. Thus, a negative initial study does not exclude perforation.

The likely mechanism of migration involves the bullet entering the cervical esophagus and subsequently progressing distally under the influence of esophageal peristalsis, demonstrating that projectiles can occasionally traverse natural anatomic conduits even in the absence of overt obstructive or severe tissue disruption^[^11^]^. This case reinforces the principle that PNIs demand a cautious and systematic approach, regardless of the patient’s initial hemodynamic stability.

According to current trauma guidelines, including ATLS and the Western Trauma Association recommendations, early evaluation must prioritize airway, breathing, and circulation, followed by a detailed assessment for “hard signs” of vascular or aerodigestive injury^[^12,13^]^. The presence of these overt or “hard signs” is summarized in Table 1 and plays a critical role in guiding urgent diagnostic and management decisions. In our patient, the absence of hallmark esophageal symptoms – such as dysphagia, odynophagia, hematemesis, hoarseness, or subcutaneous emphysema – contributed to the initially reassuring clinical picture. However, esophageal perforation remains one of the most frequently missed injuries in penetrating neck trauma and carries significant morbidity when the diagnosis is delayed^[^3^]^.

**Table 1:** Overt or “hard signs” indicative of serious injury to the major vessels or aerodigestive structures^[^12^]^.

Airway compromise	
Shock
Active bleeding
Expanding or pulsatile hematoma
Hematemesis
Massive subcutaneous emphysema/air bubbling through wound
Neurologic deficit

CTA serves as the diagnostic modality of choice for penetrating neck trauma, as it provides rapid assessment of vascular and aerodigestive structures with high accuracy. Complementary imaging methods, including contrast swallow studies or endoscopy, may be warranted when suspicion persists despite unremarkable imaging. In this case, initial AP/lateral radiographs, CTA, and barium swallow imaging demonstrated normal findings with no evidence of a leak or injury. Nonetheless, the development of fever 12 hours after admission – along with subsequent CT identification of the bullet within the stomach – promptly raised suspicion for esophageal perforation, underscoring the importance of clinical vigilance despite normal initial imaging.

Early surgical exploration and primary repair remain the cornerstone of management for cervical esophageal perforations, particularly when recognized within the early window of injury. In our patient, direct repair reinforced with an SCM muscle flap provided stable coverage and favorable postoperative healing. The subsequent spontaneous passage of the bullet through the gastrointestinal tract further highlights the unpredictable nature of missile trajectories following penetrating trauma.

Endoscopic retrieval was not pursued, as the patient remained clinically stable, and imaging confirmed intragastric migration of the projectile without evidence of obstruction, perforation, or ongoing bleeding, supporting a conservative, expectant management approach.

This case emphasizes that even clinically stable patients with initially unremarkable findings may harbor delayed or occult injuries. Timely recognition of evolving symptoms and adherence to structured diagnostic algorithms are essential for optimizing outcomes in penetrating neck trauma.

## Conclusion

This case highlights the importance of maintaining a high index of suspicion for aerodigestive tract injury in all penetrating neck wounds, even when the initial clinical evaluation and imaging appear unremarkable. Delayed esophageal perforation may remain clinically silent until secondary signs, such as fever, develop. Prompt recognition of evolving symptoms, repeat imaging, and timely surgical intervention are essential to prevent serious complications. To our knowledge, this is the first reported case of a cervical gunshot projectile migrating through the esophagus into the stomach. Vigilant monitoring and adherence to structured trauma evaluation protocols are crucial for achieving favorable outcomes in PNIs.

## Data Availability

Data are available from the corresponding author upon reasonable request.

## Patient’s perspective

The patient expressed gratitude for the prompt diagnosis and the successful surgical treatment he received. He stated that he felt relieved to have survived a potentially life-threatening injury and was satisfied with both the surgical outcome and his overall recovery.

## References

[1] Loss L Henry R White A Matsushima K Barrett C Lammers D. Penetrating neck trauma: a comprehensive review. Trauma Surgery & Acute Care Open 2025;10:e001619. 40166772 10.1136/tsaco-2024-001619 PMC11956299

[2] Neck trauma - StatPearls - NCBI bookshelf. [Accessed 24 April 2026]. https://www.ncbi.nlm.nih.gov/books/NBK470422/

[3] Sudarshan M Cassivi SD. Management of traumatic esophageal injuries. J Thorac Dis 2019;11:S172. 30906582 10.21037/jtd.2018.10.86 PMC6389559

[4] Kerwan A Al-jabir A Mathew G. Revised surgical CAse REport (SCARE) guideline: an update for the age of artificial intelligence. Prem J Sci 2025;10:2025.

[5] Duhaime OR Taylor MR Richman AP. Emesis of an enteral bullet: a rare case of bullet embolism to the thoracic esophagus. Trauma Case Rep 2021;34:100505. 34277921 10.1016/j.tcr.2021.100505 PMC8261653

[6] Migration of bullet through an uninjured gastrointestinal tract: a rare case report - global journal of medical students. Accessed 24 April 2026. https://thegjms.org/role-of-negative-pressure-wound-therapy-in-diabetic-foot-ulcers-a-case-report-2/

[7] Opasanon S Methasate A Akaraviputh T. Cervical esophageal injury from gunshot trauma: endoscopic management for complication. Thai J Surg 2010;31:86–88.

[8] Schraufnagel DP Mubashir M Raymond DP. Non-iatrogenic esophageal trauma: a narrative review. Mediastinum 2022;6:23. 36164360 10.21037/med-21-41 PMC9385875

[9] Retrospective study of detecting oesophageal injuries post neck trauma: CTA versus fluoroscopy. [Accessed 24 April 2026]. https://www.scielo.org.za/scielo.php?pid=S2078-67782024000100024&script=sci_arttext&utm_source=chatgpt.com 10.4102/sajr.v28i1.2930 PMC11447603 39363954

[10] Oesophageal injuries: position paper, WSES, 2013 | World Journal of Emergency Surgery | Springer Nature Link. Accessed 24 April 2026. https://link.springer.com/article/10.1186/1749-7922-9-9?utm_source=chatgpt.com 10.1186/1749-7922-9-9 PMC3899941 24447730

[11] Lottrup C Khan A Rangan V. Esophageal physiology-an overview of esophageal disorders from a pathophysiological point of view. Ann N Y Acad Sci 2020;1481:182–97. 32648992 10.1111/nyas.14417

[12] Sperry JL Moore EE Coimbra R. Western trauma association critical decisions in trauma: penetrating neck trauma. J Trauma Acute Care Surg 2013;75:936–40. 24256663 10.1097/TA.0b013e31829e20e3

[13] Kortbeek JB Al Turki SA Ali J. Advanced trauma life support, the evidence for change. J Trauma Acute Care Surg 2008;64:1638–50. 10.1097/TA.0b013e3181744b03 18545134

